# Pandemic grief in Poland: adaptation of a measure and its relationship with social support and resilience

**DOI:** 10.1007/s12144-021-01731-6

**Published:** 2021-04-24

**Authors:** Sebastian Skalski, Karol Konaszewski, Paweł Dobrakowski, Janusz Surzykiewicz, Sherman A. Lee

**Affiliations:** 1 Institute of Psychology, Polish Academy of Sciences, 1 Jaracza Street, 00-378 Warsaw, Poland; 2 Faculty of Education, University of Bialystok, Bialystok, Poland; 3 Institute of Psychology, Humanitas University, Sosnowiec, Poland; 4 Faculty of Philosophy and Education, Catholic University of Eichstaett-Ingolstadt, Eichstaett, Germany; 5 Faculty of Educational Sciences, Cardinal Stefan Wyszynski University in Warsaw, Warsaw, Poland; 6 Department of Psychology, Christopher Newport University, Newport News, VA USA

**Keywords:** Pandemic grief scale, Dysfunctional grief, COVID-19, Resilience, Perceived social support

## Abstract

Millions of people are mourning the death of a loved to COVID-19. According to previous studies, the circumstances of coronavirus disease-related deaths may lead to dysfunctional grief. The purpose of this study was to introduce the Polish adaptation of the Pandemic Grief Scale (PGS) as well as to assess the relationship between dysfunctional grief due to a COVID-19 death, resilience and perceived social support. The adaptation was carried out on a general population sample of 286 individuals aged 18–54 years, with the evaluation being performed on a group comprising 214 people aged 18–78 years, who lost a loved one during the pandemic. The Polish version of PGS revealed a single-factor structure with strong internal consistency (α = 0.89). The PGS scores were associated with measures of complicated grief (Inventory of Complicated Grief), depression (Kutcher Adolescent Depression Scale) and lower resilience (Resilience Scale 14), which confirmed the scale’s convergent validity. No relation between PGS scores and health behaviors (Inventory of Health Behaviors) was observed, which confirmed the scale’s discriminant validity. The results of the bootstrapping technique revealed that resilience mediates the relationship between perceived social support (Multidimensional Scale of Perceived Social Support) and dysfunctional grief (total mediation). The results of this study suggest the need for practitioners to focus on resilience-enhancing interventions and perceived social support in order to improve mental health in people who lost their loved ones during the new coronavirus pandemic.

## Introduction

In the spring of 2020, the World Health Organization announced the coronavirus SARS-CoV-2 pandemic causing COVID-19. Numerous governments have taken unprecedented measures involving social isolation, border closures and restrictions on businesses and schools. The new coronavirus has been coined the global plague of the twenty-first century, with the pandemic contributing to anxiety in people around the world (Lai et al., 2020; Lima et al., 2020). Since then, scientists have undertaken a number of studies on the psychosocial consequences of the pandemic. Reports to date have indicated elevated symptoms of post-traumatic stress disorder (PTSD), anxiety, depression, and insomnia among healthcare professionals, patients and the general population (Bo et al., 2020; S. W. Kim & Su, 2020; Salari et al., 2020; Thakur & Jain, 2020; Zhai & Du, 2020). Amerio et al. (2020) noted that poor housing is linked to increased risk of depressive symptoms during lockdown. Importantly, the unique sensory processing patterns of depressed individuals have been reported as crucial factors in determining unfavorable outcomes (Serafini et al., 2017). However, not much attention was devoted to the bereaved, who lost their loved ones to COVID-19.

Grief is considered an emotionally painful, but natural response to loss. However, it is estimated that 2–10% of the population suffers from complications in the grief process (dysfunctional grief) that hinders their ability to return to normal psychosocial functioning (Glass, 2005). It seems that, in the case of the new coronavirus pandemic, this percentage may be higher, as people with COVID-19 most often die in hospitals, without the possibility of exchanging any meaningful words with their relatives before death or having to say “goodbye” over the phone/video (Eisma & Tamminga, 2020; Kokou-Kpolou et al., 2020). In addition, funerals and burials are postponed or organized remotely, often without the presence of the deceased person’s family (Wallace et al., 2020). It should be noted that, according to previous studies, family members of patients who died in hospitals are high risk of developing prolonged grief (Wright et al., 2010). Moreover, a significant link has been demonstrated between the inability of a dying patient to say “goodbye” to their family before death and complicated grief for the family members (Otani et al., 2017). Other studies show that severe pre-loss grief symptoms, lower levels of perceived social support, lack of preparation for death and the feeling of guilt positively predict the emergence of complicated grief, as well as the severity of depression (Li et al., 2019; Lobb et al., 2010; Romero et al., 2014). These findings were also confirmed in COVID-19 reports. In the study by Hamid and Jahangir (2020), for example, participants received less in-person support, which led to mourning in isolation. The inability to perform last rites added yet another layer of grief which resulted in complicated grief among the bereaved and had an impact on their overall well-being.

The essence of complicated grief is the chronic experience of specific and intense experiences associated with the death of a loved one (Nielsen et al., 2017; Shear, 2015). Such experiences include emotional numbness, feeling emptiness or meaninglessness of life, yearning for the deceased, difficulty trusting others, trouble accepting loss, avoiding reminders of a deceased loved one, loss-related bitterness or identity confusion. (Prigerson et al., 2009). While the above symptoms are common during the initial period of mourning and do not necessarily indicate a dysfunctional reaction to loss, a significant criterion which allows for distinguishing uncomplicated from complicated grief is its intensity and the time passed since the death of a loved one (APA, 2013). It should be noted that acute mourning reactions in the early months of bereavement constitute a risk factor for prolonged mourning at a later stage (Boelen & Lenferink, 2020; Bonanno & Keltner, 1997).

Studies to date have indicated that complicated grief carries serious behavioral (e.g. agitation, withdrawal and fatigue), psychological (e.g. loneliness, depression and suicidal ideation) and physical health-related (e.g. increased risk of heart attacks, disease and mortality) consequences (Bertuccio & Runion, 2020; Stroebe et al., 2007). To date, no Polish screening tool has been developed to identify individuals who may suffer from dysfunctional mourning due to a COVID-19-related loss of a loved one. In light of the above and the fact that COVID-19 death rates continue to rise, it seems necessary to undertake research on the Polish adaptation of the Pandemic Grief Scale (PGS).

Experiencing an acute mourning reaction due to the death of a COVID-19 patient requires the development of appropriate intervention methods which could improve the functioning of individuals in the face of a global health crisis. Past reports have shown that mental resources such as resilience and perceived social support may significantly reduce the level of acute mourning as well as complicated grief (Cao et al., 2020; Schwartz et al., 2018; Vegsund et al., 2019). To the best of our knowledge, the influence of the aforementioned resources on the level of grieving response to the loss of a COVID-19 patient has not been studied yet. Such research is vital for the development of effective mental health responses to those struggling with pathological grief during this infectious disease crisis.

Resilience and perceived social support are important mechanisms that appear to work together to affect the healing process (Dumont & Provost, 1999; Ozbay et al., 2008). For example, perceived social support safeguards mental and physical health in the face of environmental threats by helping individuals to develop resilience (Luthar et al., 2000; Ozbay et al., 2008). Pinkerton and Dolan (2007) noted that social capital and perceived family support may combine coping with resilience. Some studies in the organizational context also suggest that perceived emotional and psychological support positively predict resilience and improve mental health (Bernabé & Botia, 2016). Moreover, longitudinal studies indicate that resilience mediates the relationship between perceived social support and mental health (Koelmel et al., 2017). Resilience has also been shown to mediate the impact of perceived family support both on anxiety and depression in those who have suffered from the loss of an only child (Cao et al., 2020). Therefore, it seems that resilience may mediate the relationship between perceived social support and the intensity of the dysfunctional grieving reaction in individuals who have lost loved ones to COVID-19.

The purpose of this study was to assess the psychometric properties of the Polish language version of the PGS, including factor structure, reliability and validity. Correlations between the PGS and established measures of complicated grief, depression, and resilience were analyzed to determine the convergent validity of this measure of dysfunctional grief due to a COVID-19 loss. In line with the previous results, we expected positive relationships of PGS scores with complicated grief (Wallace et al., 2020) and with the risk of depression (Liu et al., 2020). Furthermore, we expected a negative association between PGS scores and resilience (Zhai & Du, 2020). To determine the discriminant validity of PGS, we analyzed correlations between PGS scores and health behaviors. Because grief and health behaviors are not conceptually related to one another, we assumed that there would be no correlation between these variables (Zvolensky et al., 2020). The second purpose of the study was to assess the relationship between resilience, perceived social support and dysfunctional grief due to a COVID-19 death. Based on the above literature review, we hypothesized that resilience and perceived social support may be negatively related to dysfunctional grief due to a COVID-19 death. Furthermore, resilience was believed to mediate the relationship between perceived social support and dysfunctional grief.

## Materials and Methods

### Participants and Procedure

The study was conducted in November 2020 with the consent of the Ethics Committee of the Institute of Psychology, Polish Academy of Sciences. Data was collected using Google Forms. Each individual consented to anonymous participation in the study and was informed of its objectives.

- **Sample A.** The assessment of psychometric properties was performed on a general sample of 286 Polish individuals aged 18–54 *(M* = 33.62, *SD* = 7.01), 57% of whom included women. The invitation to participate in the study was distributed through social media and websites. The study also controlled demographic variables such as education (2% basic, 2% middle school, 10% vocational, 45% secondary and 41% tertiary), place of residence (12% village, 30% city up to 100,000 inhabitants, 27% city with a 100–250 thousand population, and 31% city with a population exceeding 250 thousand) and marital status (38% single, 21% married, 36% in relationships, 2% divorced and 3% widowed). No recruitment conditions needed to be fulfilled in order to participate in the study. Its procedure consisted of filling in questionnaires regarding dysfunctional grief due to a COVID-19 death, complicated grief, health behaviors, resilience and depression. In addition, participants completed a questionnaire in order to collect basic socio-demographic data.
- **Sample B.** The assessment of the relationship between resilience, perceived social support and dysfunctional grief due to a COVID-19 death was performed on a sample of 214 Polish individuals aged 18–78 *(M* = 35.95, *SD* = 13.30), 55% of whom included women. The recruitment prerequisite involved the loss of a loved one (family member or close friend) due to the coronavirus infection. Recruitment was conducted through notices in hospitals specializing in COVID-19 treatment and in emergency intervention clinics. No additional criteria had to be met in order to participate in the study. Among the participants, 17% lost a child, 25% a spouse/partner, 48% a parent and 27% another family member or friend (17% of participants lost more than one person). The average time between loss and participation in the study was 1.76 months *(SD* = 0.82). The study also examined demographic variables such as education (2% basic, 3% middle school, 5% vocational, 55% secondary and 35% tertiary), place of residence (19% village, 23% city up to 100,000 inhabitants, 25% city with a 100–250 thousand population, and 33% city with a population exceeding 250 thousand) and marital status (23% single, 26% married, 21% in relationships, 3% divorced and 27% widowed) for use as statistical controls. The study procedure consisted of filling in the questionnaires regarding dysfunctional grief due to a COVID-19 death, resilience and perceived social support. Additionally, the participants completed a socio-demographic questionnaire.

### Measures

The Pandemic Grief Scale (PGS; Lee & Neimeyer, 2020) was used to assess dysfunctional grief caused by a COVID-19 death. This mental health screener was developed on people who lost someone to COVID-19 and comprises 5 grief symptoms that are strongly associated with distress and impairment (Lee & Neimeyer, 2020). The original version of the PGS was translated into Polish by three independent translators with a high level of proficiency in English. The translations were adjusted to the final version of the scale by the authors of the present study. Next, the final version was back-translated into English by two independent translators with a high level of proficiency in English. Any differences between the original and back-translated version of PGS were discussed and amended by authors of the study and the final version of PGS was accepted by an author of the scale. The translation of the scale was carried out in accordance with accepted principles developed for the purposes of intercultural research (Geisinger, 1994), based on the original English version. Each statement in PGS (see: *Appendix 1*) refers to the experience of the past two weeks and is evaluated on a four-point scale, where 0 = “not at all” and 3 = “almost daily or daily”. As presented in the study by Lee and Neimeyer (2020), a total score equal to or greater than seven (≥7) indicates the likelihood of dysfunctional grief due to a COVID-19 death and justifies the need for further evaluation and/or treatment.

Complicated grief was measured using the *Inventory of Complicated Grief (ICG)* by Prigerson et al. (1995) in its Polish adaptation (Ludwikowska-Świeboda & Lachowska, 2019). The scale comprises 19 statements which describe the feelings and thoughts associated with the death of a loved one. Respondents express their attitude to each of them on a five-point scale, where 0 = “never” and 4 = “always”. The reliability of the scale was excellent (*α* = 0.94).

Resilience as a personality trait was measured using the *Resilience Scale 14 (RS-14)* by Wagnild and Young (1993) in its Polish adaptation (Surzykiewicz et al., 2019). It consists of 14 statements. Individuals rate each of them on a seven-point scale, where 1 = “I disagree” and 7 = “I agree”. The reliability of the scale was strong (*α* = 0.85).

Health behaviors were assessed using the *Inventory of Health Behaviors (IHB)* by Juczyński (2001). It consists of 24 statements. Each of them is rated on a five-point scale, where 1 = “never or almost never” and 5 = “almost always or always”. The reliability of the scale was strong (*α* = 0.85).

Depression was assessed using the shortened version of the *Kutcher Adolescent Depression Scale (KADS)* by Brooks et al. (2003) in its Polish adaptation (Mojs et al., 2015). The scale includes six statements, each of which is rated on a five-point scale, where 0 = “never or almost never” and 3 = “always”. The reliability of the scale was adequate (*α* = 0.82).

Perception of social support was measured using the *Multidimensional Scale of Perceived Social Support (MSPSS)* by Zimet et al. (1988) in its Polish adaptation (Buszman & Przybyła-Basista, 2017). The scale consists of 12 statements, rated on a seven-point scale, where 1 = “I strongly disagree” and 7 = “I strongly agree”. The reliability of the scale was strong (α = 0.89).

### Data Analysis

A preliminary examination of the variables was performed. Specifically, the Kolmogorov-Smirnov test was used to assess normality, while Levene’s test was used to assess homoscedasticity. The results of this examination support the application of the parametric tests that were applied in this study. Confirmatory factor analysis (CFA) with the maximum likelihood (ML) estimation was applied to assess the factor structure of the Pandemic Grief Scale. The chi-squared statistic (*χ*^*2*^) was used to assess the sample and the implied covariance matrices; however, this statistic is strongly dependent on the sample size and provides an overly conservative assessment of the model fit. The comparative fit index (*CFI*) and the goodness-of-fit index (*GFI*) were used to assess the model fit relative to a baseline model in which all variables are uncorrelated and values above 0.95 indicate good fit, while values above 0.90 are considered to indicate acceptable fit. The root-mean-square error of approximation (*RMSEA*) was also examined. Ideally, these values should be less than 0.05, but values below 0.08 are considered acceptable (Byrne, 2016; Kline, 2015). Pearson’s *r* correlation analysis and regression analysis were used to determine the relations between the variables. The mediation model was assessed using Hayes’ Process macro. The significance level was determined at *p* < .050. The effect size was assessed based on *R*^*2*^. Data analysis was conducted in IBM SPSS Statistics 26 and IBM SPSS Amos 26.

## Results

### Polish Adaptation of PGS

The mean values obtained in individual PGS statements, together with standard deviation, as well as the discriminating power of individual items are depicted in Table 1.

**Table 1:** Mean values obtained from individual PGS statements (*N* = 286)

	***M*** **(*SD*)**	***r***
Item 1	0.43 (0.80)	0.72
Item 2	0.69 (0.91)	0.76
Item 3	0.70 (0.95)	0.80
Item 4	0.49 (0.80)	0.54
Item 5	0.51 (0.87)	0.79

*r* discriminating power (correlation coefficient with overall PGS score), *** *p* ≤ .001.

#### Factor Structure of the PGS

**Fig. 1:**
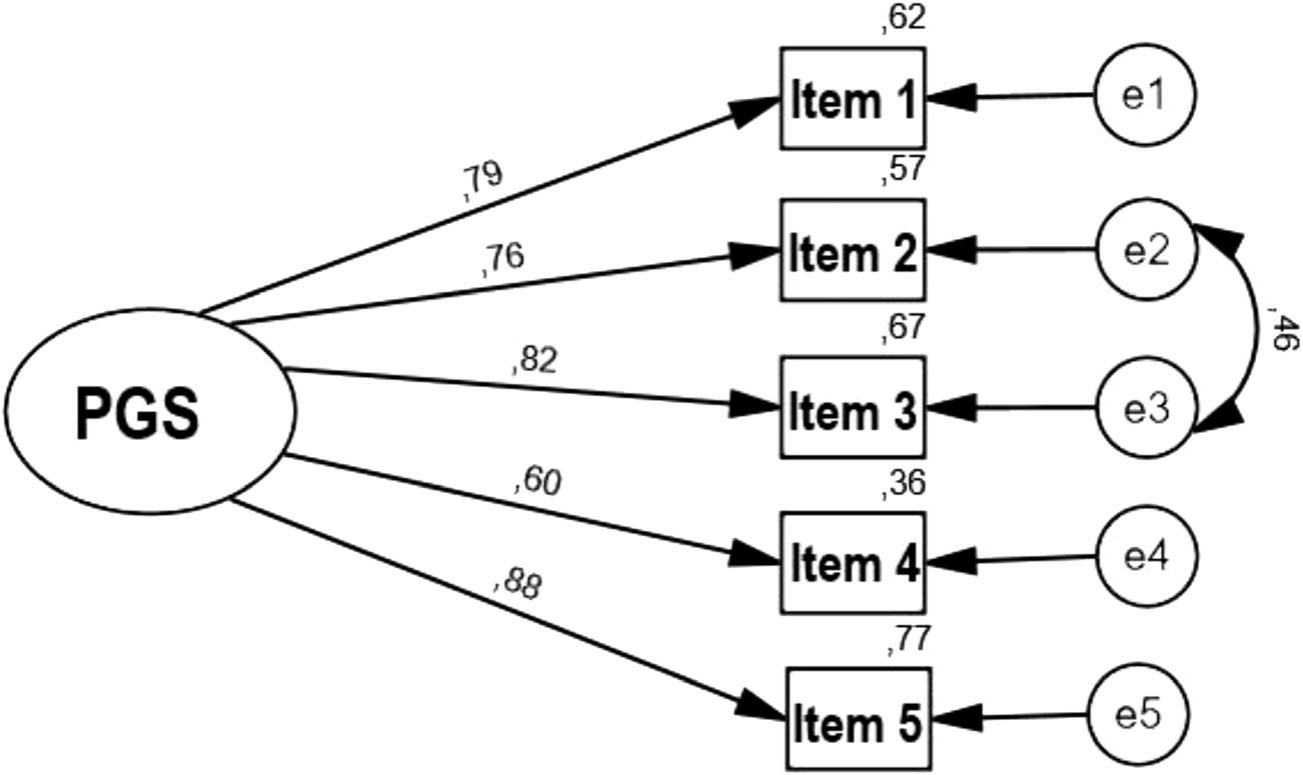
Single-factor structure of the Polish PGS version (*N* = 286)

The results of the CFA confirmed that the single-factor solution was a very good fit to the data: χ^*2*^*(4) = *5.42*; p = *.246; χ^*2*^*/df* = 1.35; *RMSEA* = 0.035 (0.000,0.102;90% *CI*); *GFI* = 0.99; *CFI* = 0.99. Factor loadings were high and exceeded a magnitude of 0.60. In the model, the modification indexes were examined and one pair of items was identified that shared the remainder of variance. Figure 1 demonstrates the standardized estimates of the confirmatory model.

#### Reliability of the PGS

Cronbach’s alpha coefficient demonstrated good reliability of the PGS, with *α* = 0.89. The composite reliability was also good, with McDonald’s omega *ω* = 0.89, and Gutmann’s λ6 *λ* = 0.88 which indicates the proportion of a scale’s variance due to a unidimensional factor.

#### Validity of the PGS

The convergent and discriminant validity of the PGS was evaluated by assessing the values of correlation coefficients with the scores of complicated grief (ICG), resilience (RS-14), health behaviors (IHB) and depression (KADS). Consistent with our convergent validity expectations, the results showed that dysfunctional grief due to a COVID-19 death (PGS) correlated positively with complicated grief (*r* = 0.76, *p* < .001) and depression (*r* = 0.50, *p* < .001), while exhibiting a negative correlation with resilience (*r* = −0.36, *p* < .001). Consistent with our discriminant validity expectation was the lack of a statistically significant correlation between dysfunctional grief due to a COVID-19 death and health behaviors (*r* = −0.09, *p* = .121). Mean values obtained in the study, as well as other correlation coefficient values are presented in Table 2.

**Table 2:** Mean values obtained in the study and correlations between variables (*N* = 286)

	***M*** **(*SD*)**	**PGS**	**ICG**	**RS-14**	**IHB**	**KADS**
Dysfunctional grief due to a COVID-19 death (PGS)	2.81 (3.59)	1	–	–	–	–
Complicated grief (ICG)	19.25 (19.06)	0.76***	1	–	–	–
Resilience (RS-14)	72.89 (14.15)	−0.36***	−0.30***	1	–	–
Health behaviors (IHB)	79.16 (16.03)	−0.09	−0.04	0.50***	1	–
Depression (KADS)	6.36 (4.82)	0.50***	0.40***	−0.56***	−0.30***	1

***p ≤ .001.

#### PGS and Demographic Variables

The analyses did not reveal statistically significant links between the PGS result and demographic data.

### PGS and Mental Resources

The mean values of dysfunctional grief due to a COVID-19 death, resilience and perceived social support, together with the standard deviation and values of correlation coefficients between variables obtained in *Sample B* are presented in Table 3. The correlation analysis showed that dysfunctional grief due to a COVID-19 death has a statistically significant negative correlation with resilience and perceived social support. In addition, resilience exhibits a statistically significant positive relationship with perceived social support. In this study, neither the time between loss and participation in the study, age, sex nor any of the other sociodemographic factors affected the results in a statistically significant way.

**Table 3:** Descriptive statistics in the study and correlations (*N* = 214)

	***M*** **(*SD*)**	**PGS**	**RS-14**	**MSPSS**
Dysfunctional grief due to a COVID-19 death (PGS)	5.51 (4.36)	1	–	–
Resilience (RS-14)	69.65 (15.39)	−0.38***	1	–
Perceived social support (MSPSS)	71.96 (9.23)	−0.34***	0.65***	1

***p ≤ .001.

**Fig. 2:**
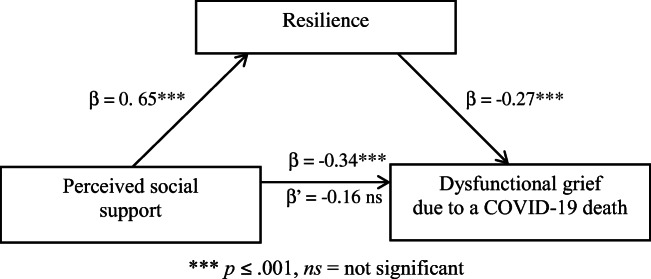
Resilience as mediator in the relationship between perceived social support and dysfunctional grief due to a COVID-19 death (*N* = 214)

Bootstrapping mediation analysis (5000) using a 95% confidence interval showed that resilience is a statistically significant mediator in the relationship between perceived social support as an independent variable and dysfunctional grief due to a COVID-19 death as a dependent variable *(indirect effect* = −0.18; −0.29,-0.06;95% *CI*; *SE* = 0.06). The overall effect equaled *β* = −0.34 (*t* = −5.20, *p* < .001; *R*^*2*^ = 0.11). The regression coefficient of the independent variable’s impact on the mediator was *β* = .0.65 (*t* = 12.33, *p* < .001; *R*^*2*^ = .0.42). The regression coefficient of the mediator’s impact on the dependent variable, with a simultaneous control of the independent variable, equaled *β* = −0.27 (*t* = −3.30, *p* = .001; *R*^*2*^ for the entire model = 0.16). Mediation explained the relation between perceived social support and dysfunctional grief due to a COVID-19 death – the direct effect equaled *β* =. -0.16 *(t* = −1.93, *p* = .055). Including an intermediate variable in the model reduced the negative link between resilience and dysfunctional grief due to a COVID-19 death to statistical insignificance, which indicates the occurrence of full mediation. Figure 2 depicts the mediation design.

## Discussion

The Polish version of the PGS revealed solid psychometric properties. The scale met the basic requirements for validity and reliability, and were in line with the results of the original PGS study (Lee & Neimeyer, 2020). For example, the results of our analysis showed that the Polish version of the PGS demonstrated good internal consistency reliability and factorial validity. In fact, the Polish version of the PGS yielded stronger reliability (*α* = 0.89) than the original PGS (*α* = 0.86) (Lee & Neimeyer, 2020). Evidence of the PGS’s convergent validity was clearly demonstrated by the strong positive correlation with an established measure of complicated grief. Moreover, the PGS score was associated with higher intensity of depression and lower resilience, which also supports the scale’s convergent validity. No relationship between the PGS and health behaviors was observed, which confirmed the measure’s discriminant validity. Taken together, the results suggest that the Polish version of the PGS could be used to measure dysfunctional grief due to a COVID-19 death for bereaved adults in Poland. Although the findings of this study suggest that the PGS may be used both in clinical practice and in research, the absolute stability of the scale is unknown, as it has not been assessed in this study. Because the severity of dysfunctional grief may change over time and also under the influence of interventions, future studies on the temporal stability of the PGS is imperative. Finally, another limitation of this study was that it was carried out on a general population (*Sample A*), which may underestimate the intensity of dysfunctional grief experienced by those in clinical samples. Therefore, future research should also replicate this psychometric research using clinical samples as well.

The results of the mediation analysis were also informative, as they were consistent with previous research. Specifically, the findings that resilience and perceived social support were correlated with each other and were associated with a lower level of dysfunctional grief, has been found in other studies (Cao et al., 2020; Schwartz et al., 2018; Vegsund et al., 2019). The mediation analysis also suggest that perceived social support and resilience may play a protective role in the mental health among the bereaved during the new coronavirus pandemic. According to Kim and Yang (2017), the co-occurrence of both the aforementioned mental resources (environmental and personality variables) is essential for optimal adaptation to traumatic events and for maintaining mental health. In addition, studies to date have observed that resilience and perceived social support influence the level of coronavirus anxiety and PTSD symptoms among healthcare professionals and the general population (Labrague & Santos, 2020; Paredes et al., 2021; Skalski et al., 2020). It is important to note, however, that this study only examined one aspect of social support, which is perceived social support. Because Cao et al. (2020) found that both perceived and objective social support are equally effective in reducing anxiety and depression among widowers, future research may be enriched by examining objective aspects of support as well. Finally, although the effect sizes were in the average range (excluding the relationship of resilience and perceived social support), the findings should still be considered important as they underscore the role of resilience and perceived social support in the non-pharmacological reduction of dysfunctional grief.

Resilience also proved to be a mediator between perceived social support and dysfunctional grief, which is consistent with previous reports (Bernabé & Botia, 2016; Cao et al., 2020; Koelmel et al., 2017; Ozbay et al., 2008). In other words, the relationship found between social support and dysfunctional grief is explained by resilience. That is, social support is linked to lower grief because it strengthens a person’s sense of resilience. Resilience is defined as the ability to maintain relatively stable, healthy mental or physical functioning during disturbing events (Block & Kremen, 1996). People with higher levels of resilience are capable of creating and maintaining constructive relationships, finding creative solutions to difficult situations and developing positive expectations about life (Hjemdal et al., 2011). Because the results of this study show that resilience is the explanatory link between social support and lower levels of dysfunctional grief, bereavement support and interventions during the pandemic should encourage safe ways the bereaved can obtain social support during their period of mourning.

The results of this study also showed interesting patterns that may be unique to the effects of the COVID-19 pandemic. For example, mean scores in Sample B regarding resilience and perceived social support proved to be lower than in previous reports found in the general population (Buszman & Przybyła-Basista, 2017; Surzykiewicz et al., 2019). Although most people show resilience in stressful situations, recent studies have shown that there is a trend towards lower resilience rates for outbreaks of infectious diseases, such as the current pandemic (Bonanno, 2004; Ferreira et al., 2020). Studies further suggest that people affected by traumatic experiences during the pandemic may overcome suffering in the longer run and become resilient (Bonanno, 2004; Bonanno et al., 2008). Lower perception of social support during the new coronavirus pandemic is a common phenomenon and stems mainly from restrictions concerning social distancing and isolation (Eisma & Tamminga, 2020). These findings highlight the challenges of providing social support during a time when social distancing measures are employed.

Another interesting finding that may also be connected to the effects of the pandemic was the lack of association between grief and time since loss. Contrary to expectations (Nielsen et al., 2017; Shear, 2015), we did not find a link between the time lapsed from loss and the severity of dysfunctional grief due to a COVID-19 death. However, Lee and Neimeyer (2020) also found no correlation between these variables. Perhaps, the short period of time that has elapsed may be too narrow to detect such a pattern. Future research would benefit from examining a longer time interval to determine if a relationship truly exists between time since loss and dysfunctional grief during the pandemic.

Notwithstanding limitations, this research reports important information regarding dysfunctional grief during the COVID-19 pandemic. Specifically, this is one of the first studies to evaluate the relationship between selected psychological resources and dysfunctional grief due to a COVID-19 death. Moreover, this is the first report of a Polish version of the Pandemic Grief Scale. The results of this study also suggest that interventions increasing resilience and perceived social support may contribute to improving the mental health of people who lost their loved ones during the new coronavirus pandemic.

## Data Availability

Not applicable.

## Appendix 1. PGS statements in Polish

Jak często w trakcie ostatnich dwóch tygodni doświadczałeś/aś poniższych myśli, uczuć lub zachowań związanych ze stratą? [Over the last 2 weeks, how often have you experienced the following thoughts, feelings, or behaviors related to your loss?]

1. Chciałem/am umrzeć aby być z osobą, która zmarła. [I wished to die in order to be with the deceased.]
2. Byłem/am zdezorientowany/a i miałem/am poczucie rozpadu mojej tożsamości z powodu straty bliskiej osoby. [I experienced confusion over my role in life or felt like my identity was diminished because of the loss.]
3. Z powodu straty bliskiej osoby nic nie miało dla mnie większego znaczenia. [Nothing seemed to matter much to me because of this loss.]
4. Trudno mi było mieć wspomnienia o osobie, która zmarła. [I found it difficult to have positive memories about the deceased.]
5. Moje życie po śmierci bliskiej osoby straciło sens, stało się puste i nie może toczyć się dalej. [I believed that without the deceased, life was either meaningless, empty, or could not go on.]
